# Longitudinal plasma phosphorylated tau 181 tracks disease progression in Alzheimer’s disease

**DOI:** 10.1038/s41398-021-01476-7

**Published:** 2021-06-12

**Authors:** Shi-Dong Chen, Yu-Yuan Huang, Xue-Ning Shen, Yu Guo, Lan Tan, Qiang Dong, Jin-Tai Yu, Alzheimer’s Disease Neuroimaging Initiative

**Affiliations:** 1 Department of Neurology and Institute of Neurology, Huashan Hospital, Shanghai Medical College, Fudan University, Shanghai, China; 2 Department of Neurology, Qingdao Municipal Hospital, Qingdao University, Qingdao, China

**Keywords:** Neuroscience, Biomarkers

## Abstract

To assess plasma phosphorylated tau181 (p-tau181) as a progression biomarker in Alzheimer’s disease (AD), we examined longitudinal plasma p-tau181 of 1184 participants (403 cognitively normal (CN), 560 patients with mild cognitive impairment (MCI), and 221 with AD dementia) from Alzheimer’s Disease Neuroimaging Initiative (ADNI). The plasma p-tau level was increased at baseline for MCI and AD dementia (mean: CN, 15.4 pg/mL; MCI, 18.4 pg/mL; AD dementia, 23.7 pg/mL; *P* < 0.001) and increased significantly over time at preclinical (Aβ-positive CN), prodromal (Aβ-positive MCI), and dementia (Aβ-positive dementia) stage of AD. A longitudinal increase of plasma p-tau181 was associated with abnormal cerebrospinal fluid biomarker levels (low Aβ42, high phosphorylated tau, and high total tau, all *P* < 0.001), amyloid accumulation (*P* < 0.001) and hypometabolism (*P* = 0.002) on positron emission tomography, atrophy in structure imaging (small hippocampal (*P* = 0.030), middle temporal (*P* = 0.008), and whole brain (*P* = 0.027) volume, and large ventricular volume (*P* = 0.008)), and deteriorated cognitive performance (global cognition and memory, language, executive function, and visuospatial function, all *P* < 0.050) at baseline. Furthermore, longitudinal plasma p-tau181 correlated with concurrent changes of nearly all these AD-related hallmarks and faster increase in plasma p-tau181 correlated with faster worsening cognition in all diagnostic groups. Importantly, most associations remained significant in Aβ-positive group and became non-significant in Aβ-negative group. Longitudinal analyses of plasma p-tau181 suggest its potential as a noninvasive biomarker to track disease progression in AD and to monitor effects of disease-modifying therapeutics in clinical trials.

## Introduction

The deposition of extracellular β-amyloid (Aβ) and accumulation of intracellular misfolded phosphorylated tau (p-tau) protein are the major hallmarks of Alzheimer’s disease (AD). Since introduced in the International Working Group (IWG)-2 criteria and the National Institute of Aging and Alzheimer Association (NIA-AA) Research Framework for the diagnosis of AD, biomarkers reflecting the two pathological features are increasingly utilized in clinical assessment of AD and in clinical trials for participants’ recruitment and outcome measures [1,2,]. At present, there are mainly two types of biomarkers for AD, namely, neuroimaging and fluid biomarkers. Nevertheless, invasiveness, limited availability, high costs, and potential side effects hamper their clinical utility and make it difficult to biologically monitor the clinical change. Thus, there is a great need for cost-effective and easily accessible biomarkers to track disease progression.

Blood immunoassays have been developed for measurement of blood tau phosphorylated at threonine 181 (p-tau181) [3–5]. Recent researches have shown promising results for plasma p-tau181 as a diagnostic biomarker of AD [6,7,]. However, it remains unclear whether plasma p-tau181 could serve as the progression biomarker for AD. Previous analyses showed that high plasma P-tau181 at baseline was associated with subsequent hippocampal atrophy, cognitive decline, and development of AD dementia from cognitively normal (CN) and mild cognitive impairment (MCI) subjects [6,7,]. Nevertheless, studies investigating longitudinal plasma p-tau181 are lacking.

In the present study, we aimed to identify the potential of plasma p-tau181 in tracking AD progression using longitudinal data. The study involved 1184 people from the Alzheimer disease neuroimaging initiative (ADNI) who were CN, or diagnosed with MCI or AD dementia. We hypothesized that plasma p-tau181 levels would increase over time with disease progression and were longitudinally associated with biochemical, imaging, and cognitive measures in the course of the disease.

## Methods

### Study design

The data used in this study was downloaded from the online repository of Alzheimer’s Disease Neuroimaging Initiative (ADNI) (http://adni.loni.usc.edu/) in July 2020. The ADNI was launched in 2003 as a public-private partnership with the primary goal of testing whether serial magnetic resonance imaging, PET, and various clinical, biologic, and neuropsychological markers can be combined to measure progression of mild cognitive impairment and early AD dementia. Regional ethical committees of all institutions approved the ADNI study. Written informed consent was obtained from all participants or authorized representatives.

### Participants

Inclusion and exclusion criteria of ADNI participants have been described previously [8]. We selected all the 1190 participants with available plasma p-tau181 recruited across 3 funding cycles (ADNI-1, ADNI-GO, and ADNI-2). Diagnostic status including CN, mild cognitive impairment (MCI), or AD dementia were based on the cognitive assessments. The CN participants reported a Mini-Mental State Examination (MMSE) score of 24 or higher and a Clinical Dementia Rating Scale (CDR) score of zero. The MCI participants reported an MMSE score of 24 or higher, objective memory loss tested by delayed recall of the Wechsler Memory Scale Logical Memory II, a CDR score of 0.5, preserved activities of daily living, and absence of dementia. The individuals considered as AD dementia fulfilled the National Institute of Neurological and Communicative Disorders and Stroke and the Alzheimer Disease and Related Disorders Association criteria for probable AD [9], reported an MMSE score between 20 and 26, and a CDR score from 0.5 to 1.0.

### Plasma phosphorylated tau 181

Plasma p-tau181 levels were measured by the Single Molecule array (Simoa) technique, using an in-house assay developed in the Clinical Neurochemistry Laboratory, University of Gothenburg, Sweden [7]. A combination of two monoclonal antibodies (Tau12 and AT270) was used to measure N-terminal to mid domain forms of P-tau181. The quantification range was from 1.0 to 128.0 pg/mL after dilution correction. The study using this assay reported within-run variations of 4.6% to 7.4% and between-run variations of 9.0% to 9.9% for the high-concentration internal quality control (iQC) samples (mean = 18.7 pg/mL, range = 16.7–20.0 pg/mL), and within-run variations of 4.6% to 12.7% and between-run variations of 12.0% to 12.7% for the low-concentration iQC samples (mean = 5.4 pg/mL, range = 4.3–6.4 pg/mL. More details of the assay can be found in the study by Karikari et al. [7].

### Biomarkers in cerebrospinal fluid

CSF was collected by lumbar punctures (LPs) in a standardized procedure as described in the ADNI procedures manual (http://adni.loni.usc.edu/). Samples were properly centrifuged, aliquoted to 500 μL in polypropylene tubes, frozen within 1 h after collection, shipped overnight on dry ice to the ADNI Biomarker Core laboratory, and stored at -80 °C. Aβ42, t-tau, and p-tau181 were measured with the corresponding Elecsys immunoassays on the Elecsys cobas e 601 analyzer as previously described [10,11,]. Meanwhile, CSF Aβ42, p-tau181, and t-tau were respectively used to define amyloid pathology (A), tau pathology (T), neurodegeneration (N) to stratify participants according to the ATN framework [2] and published cut point (CSF Aβ42 < 977 pg/mL, p-tau18 > 27 pg/mL, and t-tau>300 pg/mL) [12] were used in the definition of biomarker positivity and A/T/N status. Using two large multicentre longitudinal studies (ADNI and BioFINDER), the CSF biomarkers showed satisfying performance in predicting clinical deterioration and cut-off values for single tau biomarkers were also derived and validated [12].

### Neuroimaging

Structural brain magnetic resonance imaging (MRI) was performed using 3.0 T scanners with T1-weighted scans in volumetric magnetization-prepared rapid gradient echo (MP-RAGE) sequence. FreeSurfer, version 5.1 (FreeSurfer) was used to quantify the regional volumes according to the 2010 Desikan–Killany atlas [13]. Data of hippocampal, entorhinal, middle temporal, ventricular, and whole brain volume were used and were adjusted for total intracranial volume in our analyses.

Amyloid PET imaging in the ADNI was performed using florbetapir (18F-AV-45). The imaging data obtained from the ADNI dataset had been fully preprocessed using a standardized pipeline [14] and details of florbetapir image acquisition were stated elsewhere (adni-info.org). Briefly, florbetapir image data were obtained 50 to 70 min postinjection, and images were averaged, spatially aligned, interpolated to a standard voxel size, and smoothed to a common resolution of 8 mm full width at half maximum [15]. The MP-RAGE image of each participant from the nearest available visit was segmented and parcellated with Freesurfer (version 5.3.0) to define regions of interest (ROIs) in native space. Then, the PET images were coregistered to the corresponding MP-RAGE using SPM (version 5). The intensity-normalized standard uptake value ratio (SUVR) value for each ROI was acquired by dividing tracer uptake in these regions by the value in whole cerebellum (the predefined reference region). To estimate the global florbetapir SUVr, values from four cortical gray matter regions (frontal, anterior cingulate, precuneus, and parietal cortex) were averaged. Florbetapir-PET results were considered positive if global SUVRs were at least 1.11 as recommended by ADNI [16]. Fluorodeoxyglucose (FDG)-PET data were also obtained and reconstructed according to a standardized protocol. Briefly, FDG image data were obtained 30–60 min postinjection and underwent the same preprocessing procedure as florbetapir-PET (frames were averaged, spatially aligned, interpolated to a standard voxel size, and smoothed to a common resolution of 8 mm full width at half maximum) [15]. An FDG composite score of each scan was calculated as the mean uptake in left and right angular, temporal, and posterior cingulate regions relative to the mean of a pons and cerebellar vermis reference region.

### Determination of amyloid status

CSF Aβ42 was first used to determine amyloid status. To maximize the participants, for participants who did not have CSF assessment at baseline, CSF Aβ42 measurement within 12 months of baseline plasma p-tau181 measurement would be considered. If CSF measurement was not available, then amyloid status would be determined using PET for those who had florbetapir-PET at baseline or within 12 months of baseline plasma.

### Measurement of cognition

Global cognition was evaluated using MMSE, the 11-item version of the Alzheimer Disease Assessment Scale-Cognitive Subscale (ADAS-Cog), and the CDR Scale Sum of Boxes (CDR-SB). Specific cognitive domains were also investigated using composite measures developed by ADNI for memory, executive function (EF), language, and visuospatial (VS) function [17,18,].

### Statistical analysis

Baseline characteristics between diagnostic groups were compared using tests appropriate for the distribution of each variable including ANOVA, Kruskal–Wallis, chi-square, or Fisher’s exact test.

Linear mixed-effect (LME) models were used to estimate the longitudinal changes of plasma p-tau181 levels in groups of different clinical diagnoses (stratified by Aβ status or not) and of different ATN classification. All LME models included an group × time interaction item with random intercepts and slopes and were adjusted for age, gender, APOE ε4 counts, and education years for comparisons among groups.

Cross-sectional associations of plasma p-tau181 with CSF biomarkers’ levels, neuroimaging measures, and cognitive measures were examined using linear regressions, adjusted for age, gender, APOE ε4 counts, and education years. The same models were applied for the association between rates of plasma p-tau181 and other AD-related hallmarks at baseline. Pearson correlations were computed to investigate concurrent longitudinal changes between plasma p-tau181 and other variables with individual rates extracted from similar LME models. Rates of change for each participant were derived from these models by summing the fixed and the individual-specific random effects terms. Baseline and change rates of all variables except for the plasma p-tau181 were scaled to have zero mean and unit variance so that the effect sizes could be directly comparable between association analyses. *P* values corrected for multiple comparisons underwent Benjamini–Hochberg procedure. Association and correlation analyses were repeated in different diagnostic groups and in different amyloid status.

All statistical analyses were performed using the R statistical software (version 3.5.1). Two-sided *P* values less than 0.05 were considered statistically significant.

## Results

All ADNI participants were aged between 55 and 90 years, had acquired at least 6 years of education, and showed no significant neurological disorders other than AD. We included 1184 participants in our study after exclusion of six individuals with extreme plasma p-tau levels. The mean (SD, standard deviation) age of all the included participants was 74.3 (7.59) years; 45.7% were women; 97.8% had more than 12 years of education; 43.8% had at least one APOE ε4 allele.

The demographic, CSF biomarkers, neuroimaging, and cognition characteristics of the included participants by clinical diagnosis are shown in Table 1 and differed among diagnostic groups. Of the 1184 participants included, 403 (34.0%) participants were CN, 560 (47.8%) were MCI, and 221 (18.7%) were Dementia. The mean MMSE scores were 29.0, 28.1, and 22.1 respectively for the three clinical groups. Baseline plasma p-tau181 level differed significantly between diagnostic groups (CN, 15.1 pg/mL; MCI, 18.3 pg/mL; Dementia 24.1 pg/mL). A total of 3732 plasma p-tau181 samples were obtained up to 8 years after the baseline. Participants baseline characteristics by diagnosis and Aβ status, and by ATN profiles were presented in Appendix eTables 1–2.

**Table 1:** Baseline participant demographics.

	CN (*N* = 403)	MCI (*N* = 560)	Dementia (*N* = 221)
Age	74.9(0.33)	72.9(0.34)	75.3(0.53)
Gender	216(52.9)	238(42.4)	90(40.7)
Education	16.5(0.13)	16.1(0.12)	15.8(0.19)
APOE			
APOE ε4^−/−^, No. (%)	295(43.9)	305(45.4)	72(10.7)
APOE ε4^−/+^, No. (%)	105(25.2)	207(49.6)	105(25.2)
APOE ε4^+/+^, No. (%)	8(7.9)	49(48.5)	44(43.6)
Plasma p-tau	15.4(10.5)	18.4(11.1)	23.7(8.85)
CSF biomarkers			
Aβ42	1364.1(634.3)	1077.8(565.0)	721.0(436.0)
p-tau	22.3(9.42)	26.2(14.2)	36.8(16.1)
t-tau	242.9(91.06)	274.1(127.0)	375.1(153.2)
PET imaging			
Aβ-PET	1.11(0.17)	1.22(0.22)	1.38(0.22)
FDG-PET	1.31(0.11)	1.26(0.13)	1.06(0.15)
Structure imaging (volume)*			
Hippocampal	7395.5(937.1)	7016.8(1093.9)	5756.1(1054.5)
Entorhinal	3800.4(624.4)	3631.1(716.4)	2820.7(710.5)
Mid temporal	20305.6(2695.6)	20273.9(2719.1)	17283.1(3212.3)
Ventricular	34159.5(18634.2)	38337.5(22241.7)	50289.3(23003.5)
Whole brain	1033302.8(105601.4)	1049783.5(106082.5)	986043.3(109077.5)
Cognitive measures			
MMSE	29.0(1.23)	28.1(1.71)	22.1(3.53)
CDR-SB	0.072(0.27)	1.50(1.03)	5.34(2.46)
ADAS-Cog	5.78(3.05)	9.11(4.39)	21.9(8.22)
Memory	1.08(0.61)	0.38(0.70)	-1.00(0.59)
EF	0.85(0.82)	0.35(0.88)	-0.95(0.99)
Language	0.83(0.72)	0.32(0.76)	-0.87(1.04)
VS	0.21(0.59)	0.0058(0.71)	-0.64(1.00)
Plasma p-tau, No. of samples			
Month			
0	403	560	221
12	290	491	151
24	307	428	66
36	98	358	24
48	115	184	13
60	2	5	0
72	3	2	0
84	3	1	0
96	2	0	0

^*^Structure imaging measures reported here are unadjusted by total intracranial volume.

*CN* cognitively normal, *CSF* cerebrospinal fluid, *EF* executive function, *FDG* fluorodeoxyglucose, *MCI* mild cognitive impairment, *PET* positron emission tomography, *p-tau* phosphorylated tau, *t-tau* total tau, *MMSE* mini-mental state examination, *VS* visuospatial.

### Longitudinal plasma p-tau181 and baseline diagnosis

**Fig. 1:**
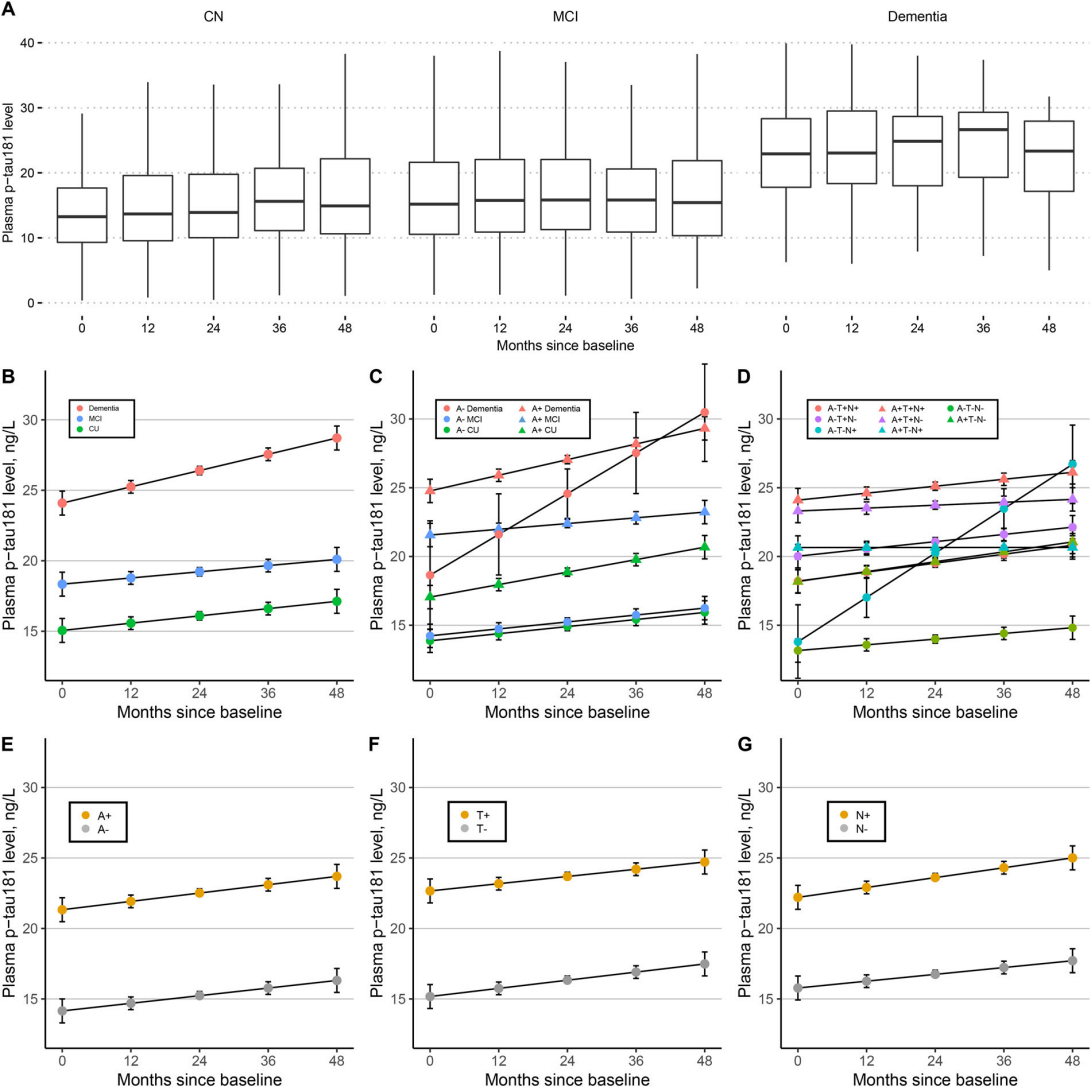
Plasma p-tau181 levels by diagnostic group, Aβ-status, and ATN classification. (**A**) Observed data in different diagnostic groups; Estimated plasma p-tau181 trajectories by diagnosis (**B**), by diagnosis and Aβ status (**C**), by ATN classification (**D**); Estimated plasma p-tau181 trajectories by A status (**E**), by T status (**F**), and by N status (**G**). *Aβ* β-amyloid; *A* amyloid; *T* tau pathology; *N* neurodegeneration; *p-tau* phosphorylated tau; *CN* cognitive normal; *MCI* mild cognitive impairment.

Results from LME models reported that all the three groups showed significant increase in plasma p-tau181 level over time (CN, 0.52 pg/mL per year; MCI, 0.45 pg/mL per year; Dementia 1.08 pg/mL per year) (Fig. 1A, B). Although no significant difference of increase rates was detected between diagnostic groups, the baseline levels differed significantly between groups (Appendix eTable 3). These results were same for the participants with Aβ-positive status (Fig. 1C, Appendix eTables 4-5). In contrast, there were no significant baseline difference between CN, MCI and dementia groups with Aβ-negative status.

Intercepts and rates were also compared within diagnostic groups, stratified by Aβ status (Appendix eTable 5). Participants with Aβ-positive status all had significantly higher baseline levels than those with Aβ-negative status within each diagnostic group. However, there were no slope differences between Aβ-negative and Aβ-positive subjects within each diagnostic group.

### Longitudinal plasma p-tau181 in groups stratified by Aβ, Tau, and Neurodegeneration

Baseline plasma p-tau181 level was higher for participants with Alzheimer’s pathologic change (A + T-N-) compared to participants with normal AD biomarkers (A-T-N-) (*P* < 0.001), and for participants with Alzheimer’s disease (A + T + N- or A + T + N + ) compared to those with Alzheimer’s pathologic change (*P* = 0.009 or *P* < 0.001) and to those with normal AD biomarkers (both *P* < 0.001) (Fig. 1D, Appendix eTables 6 and 7). However, no differences in change rates were identified between these ATN groups. Additionally, participants with A + , T + , and N + respectively showed significantly elevated plasma p-tau181 levels at baseline compared to those with A- (*P* < 0.001), T- (*P* < 0.001), and N- (*P* < 0.001) (Fig. 1E–G). Similarly, there was no slope difference between the positive biomarker group and the negative biomarker group (Appendix eTable 8).

### Association of plasma p-tau181 with CSF biomarkers, neuroimaging, and cognition

Table 2 show associations of baseline and longitudinal plasma p-tau181 levels with CSF Aβ42, p-tau181, and t-tau levels; hippocampal, entorhinal, middle temporal, ventricular, and whole brain volume; MMSE, CDR-SB, and ADAS-Cog score; memory, executive function, language, and visual-spatial composite irrespective of the diagnostic groups.

**Table 2:** Associations of baseline AD-related hallmarks with baseline and longitudinal changes of plasma p-tau181.

	Baseline plasma p-tau181				Longitudinal plasma p-tau181			
	β	s.e.	*P* value	R2	β	s.e.	*P* value	R2
CSF biomarkers								
Aβ42	−0.348	0.0472	<0.001 (<0.001)	0.312	−2.934	0.681	<0.001 (<0.001)	0.285
p-tau	0.434	0.0499	<0.001 (<0.001)	0.232	3.152	0.727	<0.001 (<0.001)	0.185
t-tau	0.381	0.0509	<0.001 (<0.001)	0.202	2.610	0.736	<0.001 (<0.001)	0.165
PET imaging								
Aβ-PET	0.494	0.0438	<0.001 (<0.001)	0.304	4.339	0.673	<0.001 (<0.001)	0.249
FDG-PET	−0.343	0.0476	<0.001 (<0.001)	0.141	−2.514	0.741	<0.001 (0.0023)	0.108
Structure imaging (volume)								
Hippocampal	−0.281	0.0450	<0.001 (<0.001)	0.277	−1.607	0.714	0.025 (0.030)	0.252
Entorhinal	−0.110	0.0484	<0.001 (<0.001)	0.092	−1.389	0.792	0.080 (0.085)	0.086
Middle temporal	−0.061	0.0484	<0.001 (<0.001)	0.091	−2.236	0.773	0.0039 (0.0083)	0.132
Ventricular	0.251	0.0448	<0.001 (<0.001)	0.261	1.963	0.662	0.0031 (0.0075)	0.246
Whole brain	−0.042	0.0495	<0.001 (<0.001)	0.050	−1.490	0.635	0.019 (0.027)	0.019
Cognitive measures								
MMSE	−0.261	0.0453	<0.001 (<0.001)	0.133	−1.047	0.671	0.12 (0.12)	0.110
CDR-SB	0.311	0.0449	<0.001 (<0.001)	0.147	1.891	0.668	0.0047 (0.0089)	0.118
ADAS-Cog	0.366	0.0442	<0.001 (<0.001)	0.174	1.770	0.664	0.0078 (0.012)	0.131
Memory composite	−0.408	0.0416	<0.001 (<0.001)	0.268	−1.773	0.631	0.0050 (0.0085)	0.212
EF composite	−0.293	0.0438	<0.001 (<0.001)	0.191	−2.558	0.648	<0.001 (<0.001)	0.172
Language composite	−0.265	0.0441	<0.001 (<0.001)	0.177	−1.458	0.653	0.026 (0.029)	0.154
VS composite	−0.238	0.0470	<0.001 (<0.001)	0.065	−1.601	0.694	0.021 (0.027)	0.048

Baseline levels of all AD-related hallmarks were normalized to have zero mean and unit variance so that effect sizes were directly comparable.

*P* values in parentheses were corrected for multiple comparisons by Benjamini–Hochberg procedure.

*Aβ* β-amyloid, *AD* Alzheimer’s disease, *ADAS-Cog* Alzheimer Disease Assessment Scale-Cognitive Subscale, *CDR-SB* Clinical Dementia Rating Scale Sum of Boxes, *CSF* cerebrospinal fluid, *EF* executive function, *FDG* fluorodeoxyglucose, *MCI* mild cognitive impairment, *PET* positron emission tomography, *p-tau* phosphorylated tau, *t-tau* total tau, *MMSE* mini-mental state examination, *VS* visuospatial.

All the variables were cross-sectionally associated with the plasma p-tau181 level at baseline and the associations remained significant after correction for multiple comparisons. All the associations remained significant in the Aβ positive group with exception of CSF Aβ42, while only CSF p-tau was significantly associated with plasma *p*-tau181 at baseline in the Aβ negative group after corrections for multiple comparisons (Appendix eTable 9).

Except for the entorhinal volume and MMSE score, all the variables were also associated with more rapid change of plasma p-tau181 levels regardless of *P* value corrections. Of the CSF biomarkers, p-tau had the strongest association with longitudinal plasma p-tau181 change. Of the imaging measures, Aβ-PET and middle temporal volume had the largest associations with plasma p-tau slope. CDR-SB, ADAS-Cog, memory composite, and EF composite had the largest associations among the cognitive measures. After dividing participants into different amyloid status, we observed that no associations were significant (all corrected P > 0.05) in the Aβ negative group (Appendix eTable 10). In contrast, the associations remained significant for all CSF biomarkers, both PET imaging measures, ventricular volume, whole brain volume, CDR-SB, and EF composite and entorhinal volume, ADAS-Cog, memory composite, language composite, and VS composite had marginally significant associations with longitudinal plasma p-tau181.

### Association of longitudinal plasma p-tau181 with CSF biomarkers, neuroimaging, and cognition in different diagnostic groups

We reported associations in each diagnostic group in Appendix eTable 11. Among CN participants, only Aβ PET was associated with the longitudinal plasma p-tau181 after *P* value correction. In MCI group, faster plasma p-tau181 increase was associated with lower CSF Aβ level, greater cerebral Aβ deposition, lower uptake in FDG-PET, larger ventricular volume, higher CDR-SB score, and lower memory, executive function, and language composites. Among participants with AD dementia, the association remained significant only for CSF p-tau181, CSF t-tau, and Aβ PET.

### Variability in plasma p-tau181 rates

**Fig. 2:**
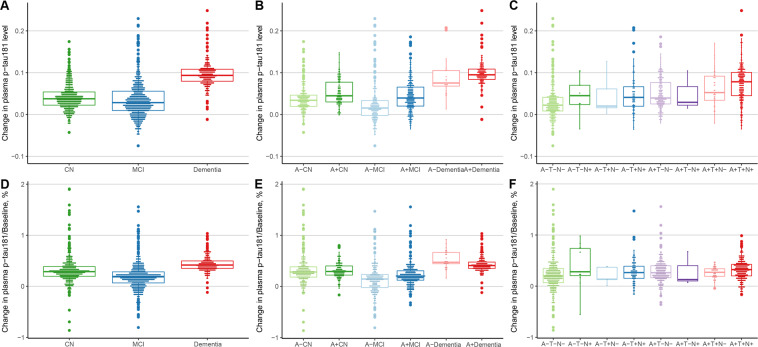
Distribution of participant-specific plasma p-tau181 slope. Participant-specific plasma p-tau181 change rate by diagnosis (**A**), by diagnosis and Aβ status (**B**), and by ATN classification (**C**); Variability of participant-specific plasma p-tau181 change rate by diagnosis (**D**), by diagnosis and Aβ status (**E**), and by ATN classification (**F**). Abbreviation: *Aβ* β-amyloid; *A* amyloid; *T* tau pathology; *N* neurodegeneration; *p-tau* phosphorylated tau; *CN* cognitive normal; *MCI* mild cognitive impairment.

Figure 2 shows the variability in participant-specific rates of plasma p-tau181. When compared between clinical diagnoses, variability of slopes was highest in the CN group (SD, 0.27% of baseline levels vs 0.26% in patients in the MCI group and 0.16% in the dementia group). When stratifying by diagnosis and Aβ status, variability was higher for Aβ-negative groups compared with Aβ-positive groups (SD, 0.16% of baseline in the Aβ-positive CN group vs 0.31% in the Aβ-negative CN group; 0.21% in the Aβ-positive MCI group vs 0.32% in the Aβ-negative MCI group; and 0.15% of baseline levels in the Aβ-positive AD group vs 0.17% in the Aβ-negative AD group). When stratified by ATN classification, slope variability in groups of Alzheimer’s pathological change and Alzheimer’s disease had lower variability compared with the group of normal AD biomarker (0.23% of baseline in A + T-N-, 0.12% of baseline in A + T + N-, and 0.20% of baseline in A + T + N + vs 0.35% in A-T-N-).

### Concurrent changes in plasma p-tau181 levels and other measures

In the whole sample, longitudinal plasma p-tau181 showed significant associations with concurrent changes of multiple AD-related hallmarks including CSF p-tau, t-tau, Aβ-PET, FDG-PET, hippocampal volume, middle temporal volume, ventricular volume, whole brain volume, MMSE, CDR-SB, ADAS-Cog, memory composite, EF composite, language composite, and visuospatial composite (Table 3). The longitudinal memory composite showed the strongest correlation with concurrent plasma p-tau181 change compared to other AD-related hallmarks.

**Table 3:** Correlations of concurrent changes in plasma p-tau181 and other measures.

	ρ	*P* value
CSF biomarkers		
Aβ42	0.0519	0.10 (0.11)
p-tau	0.1393	<0.001 (0.031)
t-tau	0.1391	<0.001 (0.028)
PET imaging		
Aβ-PET	0.1034	<0.001 (0.0011)
FDG-PET	−0.0671	0.024 (0.027)
Structure imaging (volume)		
Hippocampal	−0.0699	0.017 (0.021)
Entorhinal	−0.0014	0.96 (0.96)
Middle temporal	−0.1078	<0.001 (<0.001)
Ventricular	0.1299	<0.001 (<0.001)
Whole brain	−0.1342	<0.001 (<0.001)
Cognitive measures		
MMSE	-0.0885	0.0023 (0.0036)
CDR-SB	0.0716	0.014 (0.018)
ADAS-Cog	0.1114	<0.001 (0.009)
Memory composite	−0.1434	<0.001 (0.0017)
EF composite	−0.1293	<0.001 (<0.001)
Language composite	−0.0925	0.0014 (0.0024)
VS	−0.0835	0.0041 (0.0058)

*P* values in parentheses were corrected for multiple comparisons by Benjamini-Hochberg procedure. *Aβ* β-amyloid, *AD* Alzheimer’s disease, *ADAS-Cog* Alzheimer Disease Assessment Scale-Cognitive Subscale, *CDR-SB* Clinical Dementia Rating Scale Sum of Boxes; *CSF* cerebrospinal fluid, *EF* executive function, *FDG* fluorodeoxyglucose, *MCI* mild cognitive impairment, *PET* positron emission tomography, *p-tau* phosphorylated tau; *t-tau* total tau; *MMSE* mini-mental state examination; *VS* visuospatial.

After stratifying participants by amyloid status, we noted that all the 15 significant associations identified in the whole sample remained in the Aβ positive group except for CDR-SB and entorhinal volume and became non-significant in the Aβ negative group except for whole brain volume and memory composite (Appendix eTable 12).

Greater increase of plasma p-tau181 also correlated with the accelerated decrease of memory composite in all three diagnostic groups except that the association in MCI became marginally significant after correction for multiple comparisons (Appendix eTable 13). Greater rates of plasma p-tau181 changes were also associated with accelerated loss of ventricular volume and whole brain volume in CN controls and patients with MCI, and with accelerated increase of CSF p-tau181 and t-tau in CN controls and patients with dementia. There were also associations with Aβ PET in CN group, with executive function in MCI group, and with language and visuospatial function in dementia group.

## Discussion

The findings in the present study supported our primary hypothesis that plasma p-tau181 levels increase over time with disease progression. Compared with CN groups, plasma p-tau181 levels was increased at baseline in patients with MCI and dementia. Besides, increasing levels over time were identified at preclinical (Aβ-positive cognitively normal), prodromal (Aβ-positive MCI), and dementia (Aβ-positive dementia) stage of Alzheimer’s disease. The secondary hypothesis that plasma p-tau181 were longitudinally associated with other AD-associated measures throughout the course of the disease was also supported. Associations between longitudinal plasma p-tau181 and biochemical, imaging, and cognitive measures at baseline were extensively found and were strongest in patients with MCI. Besides, change of plasma p-tau181 correlated with change of cognitive measures in all diagnostic groups, with change of imaging measures in CN and MCI groups, and with change of CSF biomarkers in MCI and dementia groups. Importantly, all the significant associations identified in the whole sample were driven by the amyloid positive group. Taken together, our analysis of longitudinal plasma p-tau181 suggest that the plasma p-tau181 is a dynamic biomarker along the course of the disease and may be used to track disease progression in AD.

Plasma p-tau181 may outperform other biomarkers in monitoring clinical change of AD. Although MRI was recognized to show neurodegeneration in AD [19], the presence of characteristic imaging features used in clinical settings such as hippocampus atrophy and their changes only merge at the later stage of the disease. Fluid biomarkers such as plasma neurofilament lights were also identified in the previous study that was capable of tracing disease progression [20]. However, these molecules were not as specific as p-tau181 to the AD pathology [21–23]. Other neurological comorbidity may interfere with the NLF levels to index AD severity for NLF levels would rise as long as the neuronal injury appear [20]. Thus, the plasma p-tau181 is competent as an indicator for AD progression. It is suggested that, once validated to regulatory standards, this easily-accessible biomarker would prompt the development of novel disease-modifying medicine and, possibly, offer guidance in clinical decisions once such treatments become available. Besides, considering a rather close relation between the memory composite and the plasma p-tau181, it is promising that the memory function can be estimated objectively in future memory clinics. Additionally, the possible dual function of diagnosis and progression indication makes it a desirable biomarker for clinical use in primary care. For these purposes, we recommend that measurement of plasma p-tau181 should be included in future observational and therapeutic trials for AD to further investigate and validate its utility in clinical practice.

A recent work on the longitudinal plasma p-tau181 discovered that the level of plasma p-tau181 plateaued within the last four years prior to death in patients with AD neuropathology [24]. This appeared to be contradictory with our findings that plasma p-tau181 still increase at the stage of dementia. The difference may attribute to the distinct clinical severity of included subjects. While the participants included in the literature reported a mean MMSE score of 17.7 at baseline, the mean MMSE score was 22.1 for individuals with AD dementia in our study. Thus, it might be the case that the level would keep increasing until the stage of mild dementia and then plateau at the end stage of AD course. In addition, we found no difference of change rate between different diagnostic groups for plasma p-tau181. It seemed that the level of the blood biomarker increased in a linear fashion from the asymptomatic stage to mild dementia. Meanwhile, a positive association was clearly illustrated between the change rate of plasma p-tau181 and baseline AD-related outcomes such as Aβ-PET and ADAS-Cog score, which suggested that the rate of plasma p-tau181 change would be accelerated as the disease progressed. One explanation would be insufficient sample size or follow up time to detect the minor signal as annual change of plasma p-tau181 was less than 1 pg/mL. We also noticed that participants in A-T-N- and A-T-N + showed elevating levels of plasma p-tau181. The analysis of longitudinal trajectories of plasma p-tau181 by Moscoso A might explained it as they found earliest elevations of plasma p-tau181 levels occurred before PET and CSF biomarkers of amyloid reached their respective abnormality thresholds [25]. Small sample sizes of certain groups (e.g. *n* = 13 in A-T-N + ) might also had an impact on analytical results, giving an inaccurate estimate.

Our study replicated some previous findings on plasma p-tau181 [6,7,] and comprehensively revealed its associations with other AD-related biological and clinical outcomes for the first time. Besides, by first using CSF Aβ42 (higher sensitivity and better linked to disease state compared to amyloid-PET [1,26,]) as a main approach and then including florbetapir-PET and data within 12 months of baseline as an auxiliary approach to determine amyloid positivity, we maximized the participants in AD continuum as well as the whole sample size, generating a sufficiently large, prospective cohort. However, there are also several limitations to the present study. First, the participants enrolled in this research did not represent the totality of AD population. As mentioned above, the clinical severity is mild in dementia group according to the global cognitive measures. Thus, the analysis results cannot be extended to the population with moderate to severe dementia and future studies covering participants at advanced stage of AD would be necessary to examine the potential of plasma p-tau as progression biomarker. Second, ADNI has a relatively pure AD population by mainly including amnestic patients. Reproducibility of findings with different phenotypes of AD and different participants from other cohorts would be beneficial. Third, for tau pathology, only CSF tau biomarkers not tau PET were investigated. As tau PET was introduced in 2015 at the phase of ADNI-3, the baseline interval between plasma p-tau181 and tau PET was quite long (more than 6 years). It would be valuable for future researches with different study designs to include PET data and to analyze the association between different modalities of tau biomarkers. Forth, there were high drop-out rates in each diagnostic group after 5 years since baseline that lead to a rather short-term follow-up data. This might affect the estimates of longitudinal plasma p-tau181 change and its association with other variables.

In conclusion, our findings suggest that plasma p-tau181 level can be used as a noninvasive biomarker to track disease progression in AD and therefore may be candidate tool to monitor effects in trials of disease-modifying therapeutics.

## Supplementary information

SUPPLEMENTAL MATERIAL

## Data Availability

Data used in the presented study were originally from the online repository of Alzheimer’s Disease Neuroimaging Initiative (ADNI) (http://adni.loni.usc.edu/). The data generated during processing and analyzing are available from the authors upon request.

## Code availability

All scripts used in analyses are available from the authors upon request.
