# Supplementary material for: Longitudinal plasma phosphorylated tau 181 tracks disease progression in Alzheimer’s disease
Source: Transl Psychiatry. 2021 Jun 12;11:356. doi: 10.1038/s41398-021-01476-7 (PMC8197760; doi:10.1038/s41398-021-01476-7)
Supplement: Supplementary file 1 — SUPPLEMENTAL MATERIAL [file 41398_2021_1476_MOESM1_ESM.pdf]

Appendix

Content

eTable 1. Baseline participant demographics by diagnosis and Aβ status 2
eTable 2. Baseline participant demographics by ATN profiles 4
eTable 3. Baseline levels and longitudinal changes of plasma p-tau181 by clinical diagnosis. 6
eTable 4. Baseline levels and longitudinal changes of plasma p-tau181 in different diagnostic groups and Aβ status. 7
eTable 5. Comparisons of baseline levels and longitudinal changes of plasma p-tau181 in different diagnostic groups and Aβ status 7
eTable 6. Baseline levels and longitudinal changes of plasma p-tau181 by ATN classification. 8
eTable 7. Comparisons of baseline levels and longitudinal changes of plasma p-tau181 between different ATN groups 8
eTable 8. Baseline levels and longitudinal changes of plasma p-tau181 by Aβ, Tau, or Neurodegeneration. 8
eTable 9. Association between plasma p-tau181 and other measures at baseline in different amyloid status. 9
eTable 10. Association between longitudinal plasma p-tau181 and other baseline measures in different amyloid status 10
eTable 11. Association between longitudinal plasma p-tau181 and other measures at baseline across diagnostic groups 11
eTable 12. Correlations of concurrent changes in plasma p-tau181 and other measures in different amyloid status. 12
eTable 13. Correlations of concurrent changes in plasma p-tau181 and other measures across diagnostic groups 13

eTable 1. Baseline participant demographics by diagnosis and Aβ status

	A-CN(N=250)	A+CN(N=123)	A-MCI(N=235)	A+MCI(N=291)	A-Dementia(N=27)	A+Dementia(N=169)
Age	74.3(6.76)	75.2(6.27)	71.1(8.13)	73.9(7.38)	77.7(7.65)	74.7(7.57)
Female sex, No. (%)	103(52.0)	58(57.4)	105(50.5)	95(37.4)	3(15.0)	60(43.2)
Education, year	16.5(2.65)	16.4(2.56)	16.2(2.54)	16.0(2.85)	16.7(2.32)	15.7(2.74)
**APOE**						
APOE ε4-/-, No. (%)	160(80.8)	54(53.5)	156(75.0)	89(35.0)	16(80.0)	37(26.6)
APOE ε4+/-, No. (%)	37(18.7)	40(39.6)	49(23.6)	122(48.0)	3(15.0)	69(49.6)
APOE ε4+/+, No. (%)	1(0.5)	7(6.9)	3(1.4)	43(16.9)	1(5.0)	33(23.7)
Plasma p-tau, pg/mL	14.2(10.1)	17.3(8.53)	14.2(9.52)	21.7(11.5)	18.3(12.11)	24.3(7.59)
**CSF biomarkers**						
Aβ42, pg/mL	1702.5(502.9)	703.6(192.0)	1567.2(488.4)	676.7(168.6)	1642.8(599.6)	588.3(165.2)
p-tau, pg/mL	8.08(0.58)	11.4(1.14)	10.7(0.74)	15.2(0.96)	16.3(3.65)	15.9(1.35)
t-tau, pg/mL	241.1(85.7)	244.9(100.2)	236.8(100.0)	303(137.7)	336.1(162.8)	380.8(151.6)
**PET imaging**						
Aβ-PET	1.04(0.11)	1.26(0.19)	1.05(0.12)	1.34(0.20)	1.03(0.15)	1.43(0.18)
FDG-PET	1.32(0.11)	1.28(0.11)	1.31(0.12)	1.22(0.13)	1.18(0.18)	1.05(0.14)
**Structure imaging (volume)***						
Hippocampal, mm^3	7445.5(962.8)	7354.4(843.2)	7337.4(1139.0)	6764.9(1000.6)	5530.0(1454.9)	5838.8(959.2)
Entorhinal, mm^3	3818.0(648.2)	3768.0(546.0)	3729.5(709.5)	3549.0(720.4)	3122.4(802.0)	2816.5(682.3)
Mid temporal, mm^3	20477.8(2710.8)	20230.5(2489.0)	20686.4(2633.1)	19959.0(2759.9)	18599.5(2724.0)	17346.1(3166.6)
Ventricular, mm^3	31589.0(15632.4)	39360.8(22979.6)	32827.9(19965.5)	42921.7(23402.4)	46624.9(22769.5)	51169.3(23784.8)
Whole brain, mm^3	1038807.3(108427.9)	1036167.1(94303.7)	1055963.0(107969.5)	1048877.1(103162.3)	990299.0(108411.0)	993731.4(119231.8)
**Cognitive measures**						
MMSE	29.1(1.20)	29.0(1.33)	28.5(1.50)	27.7(1.82)	23.0(3.41)	22.3(3.36）
CDR-SB	0.054(0.21)	0.12(0.33)	1.27(0.78)	1.66(1.12)	4.73(2.21)	5.17(2.37)
ADAS-Cog	5.71(3.03)	6.07(3.08)	7.58(3.61)	10.5(4.56)	17.9(7.05)	21.9(8.19)
Memory	1.11(0.57)	0.97(0.64)	0.67(0.66)	0.11(0.62)	-0.77(0.63)	-0.99(0.56)
EF	0.96(0.82)	0.63(0.80)	0.64(0.83)	0.12(0.84)	-0.71(1.04)	-0.93(0.94)
Language	0.84(0.72)	0.75(0.70)	0.52(0.75)	0.17(0.74)	-0.58(0.99)	-0.83(1.00)
VS	0.22(0.58)	0.19(0.60)	0.12(0.68)	-0.11(0.73)	-0.45(0.81)	-0.60(1.01)

**Plasma p-tau, No. of samples**						
Month						
0	250	123	235	261	27	169
12	198	90	210	219	16	119
24	215	92	190	173	6	49
36	53	28	168	72	3	16
48	72	35	98	0	2	9
60	0	0	3	0	0	0
72	2	0	2	0	0	0
84	3	0	0	0	0	0
96	1	0	0	0	0	0

Continuous variables were expressed as mean (SD) and categorical variables as number (%). Abbreviation: CN = cognitively normal; CSF = cerebrospinal fluid; EF = executive function; FDG = fluorodeoxyglucose; MCI = mild cognitive impairment; PET = positron emission tomography; p-tau = phosphorylated tau; t-tau = total tau; MMSE = mini-mental state examination; VS = visuospatial;

*Structure imaging measures reported here are unadjusted by total intracranial volume.

eTable 2. Baseline participant demographics by ATN profiles

	A-T-N-(N=331)	A-T-N+(N=13)	A-T+N-(N=5)	A-T+N+(N=77)	A+T-N-(N=223)	A+T-N+(N=3)	A+T+N-(N=41)	A+T+N+(N=227)
Age	71.6(7.42)	74.0(8.50)	74.7(7.38)	75.3(7.27)	73.2(7.17)	81.9(9.94)	75.9(7.25)	74.1(7.14)
Female sex, No. (%)	161(48.6)	9(69.2)	2(40.0)	39(50.6)	82(36.8)	1(33.3)	10(24.4)	120(52.9)
Education, year	16.3(2.58)	14.9(2.82)	17.8(2.28)	16.6(2.36)	16.4(2.69)	16.3(6.35)	16.4(2.58)	15.7(2.67)
**APOE**								
APOE ε4-/-, No. (%)	269(81.3)	11(84.6)	4(80.0)	48(62.3)	100(44.8)	1(33.3)	16(39.0)	63(27.8)
APOE ε4+/-, No. (%)	59(17.8)	2(15.4)	1(20.0)	27(35.1)	94(42.2)	2(66.7)	17(41.5)	118(52.0)
APOE ε4+/+, No. (%)	3(0.9)	0(0.0)	0(0.0)	2(2.6)	29(13.0)	0(0.0)	8(19.5)	46(20.3)
Plasma p-tau, pg/mL	13.3(8.78)	17.2(16.4)	21.6(10.1)	23.5(50.8)	18.1(9.80)	19.8(3.79)	23.6(11.5)	24.3(10.9)
**CSF biomarkers**								
Aβ42, pg/mL	1598.2(423.9)	1890.7(533.4)	1324.9(569.4)	1758.9(732.0)	657.5(189.3)	598.8(333.1)	592.2(161.3)	670.1(165.2)
p-tau, pg/mL	17.7(4.27)	25.6(1.09)	28.4(0.87)	37.6(12.6)	18.6(5.21)	23.2(5.77)	29.0(1.76)	44.0(13.2)
t-tau, pg/mL	203.1(46.5)	311.4(11.6)	288.5(6.27)	404.7(108.4)	198.5(52.0)	319.9(15.4)	281.5(15.4)	432.9(120.8)
**PET imaging**								
Aβ-PET	1.03(0.08)	1.04(0.09)	1.25(0.17)	1.15(0.19)	1.26(0.20)	1.42(0.19)	1.43(0.17)	1.44(0.17)
FDG-PET	1.33(0.11)	1.32(0.17)	1.30(0.04)	1.26(0.13)	1.23(0.15)	1.18(0.15)	1.20(0.13)	1.15(0.16)
**Structure imaging (volume)***								
Hippocampal, mm^3	7441.8(1062.6)	6455.4(1312.9)	7019.3(1621.9)	7034.4(1291.5)	7046.4(1051.2)	5599.0(94.8)	6663.3(887.0)	6333.6(1059.4)
Entorhinal, mm^3	3821.1(662.3)	3539.3(501.8)	3770.5(499.6)	3584.3(814.1)	3622.2(718.0)	3986.3(1387.5)	3477.2(749.4)	3241.3(762.5)
Mid temporal, mm^3	20734.1(2701.0)	19451.6(2149.0)	21243.0(4084.0)	19982.8(2463.6)	20549.6(2618.6)	18801.0(4478.9)	19956.7(2984.9)	18410.0(3103.7)
Ventricular, mm^3	31382.5(16784.6)	32422.0(23927.0)	32582.0(12839.9)	31191.3(16858.2)	47799.0(25409.8)	48717.3(33871.7)	50927.3(22266.9)	38746.5(19207.7)
Whole brain, mm^3	1054183(108762)	1018169(99059)	1052187(117408)	1031469(110363)	1059295(103121)	1017311(157467)	1062114(115543)	1012343(105292)
**Cognitive measures**								
MMSE	28.6(1.58)	28.1(2.18)	29.2(0.84)	28.1(2.14)	27.5(2.81)	25.7(5.13)	26.5(3.46)	25.5(3.40)
CDR-SB	0.80(1.17)	1.35(2.26)	1.30(0.76)	0.92(1.30)	1.56(1.80)	2.17(2.89)	2.51(2.52)	2.91(2.40)
ADAS-Cog	6.87(3.82)	8.26(6.44)	7.60(2.61)	7.92(4.82)	10.2(6.45)	14.0(8.66)	13.2(8.65)	15.3(8.41)
Memory	0.89(0.69)	0.69(0.76)	0.63(0.80)	0.63(0.82)	0.28(0.82)	-0.09(0.62)	-0.07(0.81)	-0.32(0.89)
EF	0.82(0.86)	0.60(0.86)	0.68(0.68)	0.59(0.96)	0.21(1.01)	-0.04(1.82)	-0.11(0.98)	-0.32(1.01)
Language	0.65(0.77)	0.63(0.69)	0.39(0.76)	0.58(0.82)	0.31(0.89)	0.57(1.11)	-0.13(0.89)	-0.25(0.97)
VS	0.16(0.65)	-0.08(0.88)	0.32(0.85)	0.07(0.67)	-0.03(0.78)	0.14(0.98)	-0.31(0.72)	-0.31(0.93)

**Plasma p-tau, No. of samples**								
Month								
0	331	13	5	77	223	3	41	227
12	264	8	5	58	179	3	30	12
24	262	9	5	58	147	3	24	24
36	150	3	4	27	84	1	16	80
48	114	5	4	24	49	0	8	36
60	3	0	2	0	0	0	0	0
72	3	1	0	0	0	0	0	0
84	3	1	0	0	0	0	0	0
96	0	1	0	0	0	0	0	0

Continuous variables were expressed as mean (SD) and categorical variables as number (%). Abbreviation: CN = cognitively normal; CSF = cerebrospinal fluid; EF = executive function; FDG = fluorodeoxyglucose; MCI = mild cognitive impairment; PET = positron emission tomography; p-tau = phosphorylated tau; t-tau = total tau; MMSE = mini-mental state examination; VS = visuospatial;

*Structure imaging measures reported here are unadjusted by total intracranial volume.

eTable 3. Baseline levels and longitudinal changes of plasma p-tau181 by clinical diagnosis.

	CN	MCI	Dementia	CN vs MCI	CN vs Dementia	MCI vs Dementia
**Intercept**	15.1	18.3	24.1	-2.39	-6.54	-3.70
**Intercept, p-value**	**<0.001**	**<0.001**	**<0.001**	**<0.001(<0.001)**	**<0.001(<0.001)**	**<0.001 (<0.001)**
**Slope**	0.52	0.45	1.08	0.060	-0.58	-0.64
**Slope, p-value**	**0.003(0.005)**	**0.001(0.003)**	**0.004(0.004)**	0.783(0.783)	0.188(0.282)	0.131(0.393)

Linear mixed-effect models showing effects of clinical diagnosis on plasma p-tau181 at baseline (intercept, pg/mL) and over time (slope, pg/mL per month). Comparisons were adjusted for age, gender, APOE4 status, and educational years. P-values are reported together with P-values adjusted for multiple comparisons (Benjamini-Hochberg). Abbreviations: CN = cognitively normal; MCI = mild cognitive impairment;

eTable 4. Baseline levels and longitudinal changes of plasma p-tau181 in different diagnostic groups and Aβ status.

	A-CN	A+CN	A-MCI	A+MCI	A-Dementia	A+Dementia
**Intercept**	13.870	17.047	14.230	21.560	18.643	24.767
**P**	**<0.001**	**<0.001**	**<0.001**	**<0.001**	**<0.001**	**<0.001**
**Slope**	0.043	0.075	0.041	0.035	0.242	0.092
**P**	**0.021(0.025)**	**0.007(0.040)**	**0.012(0.037)**	**0.036(0.036)**	**0.014(0.028)**	**0.017(0.026)**

Linear mixed-effect models showing effects of diagnosis and Aβ status on plasma p-tau181 at baseline (intercept, pg/mL) and over time (slope, pg/mL per month).
Abbreviations: A+ = positive β-amyloid status; A-= negative β-amyloid status; CN = cognitively normal; MCI = mild cognitive impairment;

eTable 5. Comparisons of baseline levels and longitudinal changes of plasma p-tau181 in different diagnostic groups and Aβ status

	Within A+ goups			Within A- groups			Within each diagnostic group		
	**A+Dementia vs A+MCI**	**A+Dementia vs A+CN**	**A+MCI vs A+CN**	**A-Dementia vs A-MCI**	**A-Dementia vs A-CN**	**A-MCI vs A-CN**	**A+Dementia vs A-Dementia**	**A+MCI vs A-MCI**	**A+CN vs A-CN**
**Intercept**	2.609	6.672	4.063	2.611	3.582	0.972	5.321	5.323	2.231
**P values**	**0.004(0.010)**	**<0.001(<0.001)**	**<0.001(<0.001)**	0.178(0.200)	0.063(0.081)	0.250(0.250)	**0.008(0.0143)**	**<0.001(<0.001)**	**0.031(0.047)**
**Slope**	0.057	0.017	-0.040	0.201	0.199	-0.002	-0.151	-0.006	0.032
**P values**	0.174(0.391)	0.723(0.929)	0.212(0.381)	0.043(0.392)	0.047(0.210)	0.936(0.936)	0.154(0.461)	0.786(0.884)	0.340(0.510)

Comparisons between groups were derived from the linear mixed-effect models adjusting for age, gender, APOE4 status, and educational years. P-values are reported together with P-values adjusted for multiple comparisons (Benjamini-Hochberg). Abbreviations: A+ = positive β-amyloid status; A-= negative β-amyloid status; CN = cognitively normal; MCI = mild cognitive impairment.

eTable 6. Baseline levels and longitudinal changes of plasma p-tau181 by ATN classification.

	A-T-N-	A+T-N-	A+T+N-	A+T+N+
**Intercept**	13.160	18.180	23.307	24.100
**P**	**<0.001(<0.001)**	**<0.001(<0.001)**	**<0.001(<0.001)**	**<0.001(<0.001)**
**Slope**	0.035	0.060	0.017	0.042
**P**	**0.011(0.022)**	**0.002(0.008)**	0.723(0.723)	**0.039**(0.052)

Linear mixed-effect models showing plasma p-tau181 at baseline (intercept, pg/mL) and over time (slope, pg/mL per month) in ATN groups of normal AD biomarkers (A-T-N-), Alzheimer's pathologic change (A+T-N-), and Alzheimer's disease (A+T+N- and A+T+N+). Abbreviations: AD = Alzheimer's disease; A+ = positive β-amyloid status; A-= negative β-amyloid status.

eTable 7. Comparisons of baseline levels and longitudinal changes of plasma p-tau181 between different ATN groups

	A+T-N- vs A-T-N-	A+T+N- vs A-T-N-	A+T+N+ vs A-T-N-	A+T+N- vs A+T-N-	A+T+N+ vs A+T-N-	A+T+N+ vs A+T+N-
**Intercept**	3.547	7.674	8.974	4.127	5.426	1.300
**P**	**<0.001(<0.001)**	**<0.001(<0.001)**	**<0.001(<0.001)**	**0.007(0.009)**	**<0.001(<0.001)**	0.399(0.399)
**Slope**	0.025	-0.017	0.007	-0.043	-0.018	0.025
**P**	0.309(1.000)	0.732(0.879)	0.782(0.782)	0.422(1.000)	0.542(1.000)	0.646(0.968)

Comparisons between groups were derived from the linear mixed-effect models adjusting for age, gender, APOE4 status, and educational years. P-values are reported together with P-values adjusted for multiple comparisons (Benjamini-Hochberg). Abbreviations: A = β-amyloid status; T = tau status; N = neurodegeneration status.

eTable 8. Baseline levels and longitudinal changes of plasma p-tau181 by Aβ, Tau, or Neurodegeneration.

	A-	A+	A+ vs A-	T-	T+	T+ vs T-	N-	N+	N+ vs N-
**Intercept**	14.150	21.330	5.110	15.172	22.663	5.763	15.777	22.209	4.875
**P**	**<0.001**	**<0.001**	**<0.001**	**<0.001**	**<0.001**	**<0.001**	**<0.001**	**<0.001**	**<0.001**
**Slope**	0.045	0.049	0.004	0.048	0.043	-0.005	0.040	0.058	0.017
**P**	**0.001**	**0.001**	0.828	**0.000**	**0.012**	0.793	**0.001**	**0.001**	0.406

Comparisons between groups were derived from the linear mixed-effect models adjusting for age, gender, APOE4 status, and educational years. Abbreviations: A = β-amyloid status; T = tau status; N = neurodegeneration status.

eTable 9. Association between plasma p-tau181 and other measures at baseline in different amyloid status

	A-				A+			
	β	s.e.	P value	R2	β	s.e.	P value	R2
**CSF biomarkers**								
Aβ42	-0.029	0.074	0.697(0.741)	0.028	-0.127	0.091	0.161(0.161)	0.114
p-tau	0.199	0.071	**0.005**(**0.043**)	0.117	0.693	0.085	**<0.001(<0.001)**	0.217
t-tau	0.185	0.070	**0.009**(0.051)	0.121	0.639	0.086	**<0.001(<0.001)**	0.207
**PET imaging**								
Aβ-PET	0.140	0.066	**0.036**(0.151)	0.070	0.683	0.082	**<0.001(<0.001)**	0.177
FDG-PET	-0.059	0.066	0.367(0.521)	0.098	-0.595	0.086	**<0.001(<0.001)**	0.095
**Structure imaging (volume)**								
Hippocampal	-0.116	0.063	0.067(0.19)	0.264	-0.402	0.084	**<0.001(<0.001)**	0.202
Entorhinal	-0.112	0.073	0.128(0.242)	0.043	-0.433	0.090	**<0.001(<0.001)**	0.099
Middle temporal	-0.014	0.069	0.843(0.843)	0.160	-0.478	0.090	**<0.001(<0.001)**	0.093
Ventricular	0.112	0.065	0.083(0.176)	0.211	0.277	0.082	**0.001(0.001)**	0.203
Whole brain	-0.028	0.058	0.632(0.716)	0.350	-0.360	0.081	**<0.001(<0.001)**	0.211
**Cognitive measures**								
MMSE	-0.122	0.067	0.067(0.163)	0.026	-0.372	0.084	**<0.001(<0.001)**	0.091
CDR-SB	0.044	0.067	0.514(0.672)	0.021	0.533	0.083	**<0.001(<0.001)**	0.124
ADAS-Cog	0.129	0.066	0.05(0.17)	0.053	0.534	0.083	**<0.001(<0.001)**	0.115
Memory composite	-0.179	0.062	0.004(0.075)	0.145	-0.584	0.080	**<0.001(<0.001)**	0.182
EF composite	-0.080	0.063	0.206(0.318)	0.121	-0.457	0.083	**<0.001(<0.001)**	0.117
Language composite	-0.031	0.064	0.626(0.76)	0.117	-0.451	0.082	**<0.001(<0.001)**	0.131
VS composite	-0.096	0.067	0.15(0.256)	0.020	-0.276	0.087	**0.002(0.002)**	0.038

Baseline levels of all other measures were normalized to have zero mean and unit variance so that effect sizes were directly comparable.
P values in parentheses are corrected for multiple comparisons by Benjamini-Hochberg procedure.
P values less than 0.05 were shone in bold.
Abbreviation: Aβ = β-amyloid; AD = Alzheimer's disease; ADAS-Cog = Alzheimer Disease Assessment Scale-Cognitive Subscale; CDR-SB = Clinical Dementia Rating Scale Sum of Boxes; CSF = cerebrospinal fluid; EF = executive function; FDG = fluorodeoxyglucose; MCI = mild cognitive impairment; PET = positron emission tomography; p-tau = phosphorylated tau; t-tau = total tau; MMSE = mini-mental state examination; VS = visuospatial

eTable 10. Association between longitudinal plasma p-tau181 and other baseline measures in different amyloid status

	A-				A+			
	β	s.e.	P value	R2	β	s.e.	P value	R2
**CSF biomarkers**								
Aβ42	-1.879	1.125	0.096(0.406)	0.034	-2.448	1.096	**0.026(0.049)**	0.120
p-tau	2.227	1.080	**0.040**(0.676)	0.109	3.364	1.091	**0.002(0.018)**	0.128
t-tau	2.061	1.077	0.056(0.319)	0.115	2.732	1.088	**0.012(0.03)**	0.133
**PET imaging**								
Aβ-PET	2.282	1.165	0.051(0.431)	0.069	4.217	0.986	**<0.001(<0.001)**	0.102
FDG-PET	-0.559	1.150	0.627(0.97)	0.096	-2.783	1.056	**0.009(0.049)**	0.028
**Structure imaging (volume)**								
Hippocampal	-0.645	1.100	0.558(1.185)	0.259	-1.513	1.064	0.156(0.166)	0.168
Entorhinal	0.378	1.235	0.760(0.994)	0.038	-2.209	1.155	0.056(0.074)	0.061
Middle temporal	-1.920	1.151	0.096(0.327)	0.165	-2.315	1.165	**0.047**(0.081)	0.046
Ventricular *	-0.559	1.150	0.627(1.067)	0.096	-2.783	1.056	**0.009(0.037)**	0.028
Whole brain	-0.712	0.870	0.414(1.005)	0.350	-2.611	1.014	**0.01(0.029)**	0.191
**Cognitive measures**								
MMSE	-0.607	1.048	0.563(1.063)	0.020	-0.728	1.001	0.468(0.468)	0.061
CDR-SB	0.265	1.048	0.800(0.907)	0.020	2.594	0.997	**0.009(0.032)**	0.072
ADAS-Cog	0.035	1.039	0.973(0.973)	0.046	1.967	1.003	**0.050**(0.078**)**	0.058
Memory composite	-0.474	0.987	0.631(0.894)	0.132	-1.749	0.975	0.073(0.083)	0.111
EF composite	-1.261	0.993	0.205(0.580)	0.121	-2.464	0.991	**0.013(0.028)**	0.081
Language composite	0.159	0.996	0.873(0.928)	0.116	-1.785	0.985	0.071(0.086)	0.091
VS composite	-0.292	1.050	0.781(0.948)	0.017	-1.983	1.019	0.052(0.074)	0.027

Baseline levels of all other measures were normalized to have zero mean and unit variance so that effect sizes were directly comparable.
P values in parentheses are corrected for multiple comparisons by Benjamini-Hochberg procedure.
P values less than 0.05 were shone in bold.
Abbreviation: Aβ = β-amyloid; AD = Alzheimer's disease; ADAS-Cog = Alzheimer Disease Assessment Scale-Cognitive Subscale; CDR-SB = Clinical Dementia Rating Scale Sum of Boxes; CSF = cerebrospinal fluid; EF = executive function; FDG = fluorodeoxyglucose; MCI = mild cognitive impairment; PET = positron emission tomography; p-tau = phosphorylated tau; t-tau = total tau; MMSE = mini-mental state examination; VS = visuospatial

eTable 11. Association between longitudinal plasma p-tau181 and other measures at baseline across diagnostic groups

	CN				MCI				Dementia			
	β	s.e.	P value	R2	β	s.e.	P value	R2	β	s.e.	P value	R2
**CSF biomarkers**												
Aβ42	-2.674	1.309	0.042(0.357)	0.150	-2.799	0.900	**0.00(0.017)**	0.309	-2.172	2.297	0.346(0.588)	0.150
p-tau	2.491	1.335	0.063(0.357)	0.117	1.599	0.973	0.101(0.143)	0.194	7.949	2.284	**0.001(0.011)**	0.159
t-tau	2.420	1.343	0.073(0.309)	0.106	0.787	0.981	0.423(0.423)	0.179	7.002	2.311	**0.003(0.024)**	0.139
**PET imaging**												
AV45	5.785	1.482	**0.001(0.002)**	0.177	3.107	0.845	**<0.001(0.004)**	0.247	5.834	2.027	**0.005(0.025)**	0.189
FDG-PET	0.791	1.588	0.619(0.877)	0.044	-2.372	0.926	**0.011(0.037)**	0.107	-2.989	2.357	0.207(0.502)	0.018
**Structure imaging**												
Hippocampus	0.778	1.387	0.575(0.889)	0.250	-1.415	0.911	0.121(0.158)	0.260	1.881	2.204	0.395(0.610)	0.223
Entorhinal	-0.131	1.565	0.933(0.933)	0.047	-0.950	1.034	0.359(0.436)	0.044	-2.513	2.575	0.331(0.625)	0.021
Mid temporal	0.961	1.465	0.512(0.870)	0.180	-1.690	0.985	0.087(0.134)	0.134	3.423	2.463	0.167(0.473)	0.097
Ventricular volumn	0.163	1.172	0.889(0.945)	0.209	2.336	0.855	**0.006(0.037)**	0.302	-0.021	2.272	0.993(0.993)	0.151
Whole brain	0.280	1.037	0.787(0.956)	0.368	-1.723	0.823	0.037(0.070)	0.332	0.625	2.316	0.787(0.956)	0.095
**Cognitive measures**												
MMSE	1.840	1.236	0.137(0.334)	0.048	-0.745	0.899	0.408(0.433)	0.097	-0.838	2.151	0.697(0.911)	-0.002
CDR-SB	-2.148	1.260	0.089(0.303)	0.013	2.419	0.936	**0.010(0.043)**	0.023	3.678	2.080	0.079(0.267)	0.066
ADAS-Cog	1.840	1.192	0.123(0.350)	0.112	0.779	0.908	0.391(0.444)	0.087	2.457	2.148	0.254(0.540)	0.006
Memory composite	0.307	1.149	0.790(0.895)	0.179	-2.045	0.848	**0.016(0.046)**	0.197	-0.379	2.144	0.860(0.914)	0.007
EF composite	-1.164	1.177	0.323(0.687)	0.136	-2.038	0.847	**0.016(0.040)**	0.200	-5.006	2.119	0.019(0.081)	0.030
Language composite	0.878	1.202	0.466(0.880)	0.101	-2.011	0.853	**0.018(0.040)**	0.188	-0.424	2.103	0.841(0.953)	0.045
VS composite	0.463	1.255	0.712(0.931)	0.018	-1.774	0.927	0.056(0.096)	0.040	-1.543	2.154	0.475(0.672)	-0.002

Baseline levels and rates of change of all other measures were normalized to have zero mean and unit variance so that effect sizes were directly comparable. P values in parentheses are corrected for multiple comparisons by Benjamini-Hochberg procedure. Abbreviation: Aβ = β-amyloid; AD = Alzheimer's disease; ADAS-Cog = Alzheimer Disease Assessment Scale-Cognitive Subscale; CDR-SB = Clinical Dementia Rating Scale Sum of Boxes; CSF = cerebrospinal fluid; EF = executive function; FDG = fluorodeoxyglucose; MCI = mild cognitive impairment; PET = positron emission tomography; p-tau = phosphorylated tau; t-tau = total tau; MMSE = mini-mental state examination; VS = visuospatial

eTable 12. Correlations of concurrent changes in plasma p-tau181 and other measures in different amyloid status.

	A-		A+	
	ρ	P value	ρ	P value
**CSF biomarkers**				
Aβ42	0.053	0.263(0.498)	0.036	0.417(0.472)
p-tau	0.060	0.205(0.436)	0.173	**<0.001(0.001)**
t-tau	0.074	0.118(0.288)	0.172	**<0.001(0.001)**
**PET imaging**				
Aβ-PET	0.027	0.546(0.714)	0.088	**0.037(0.049)**
FDG-PET	0.024	0.587(0.713)	-0.123	**0.003(0.013)**
**Structure imaging (volume)**				
Hippocampal	-0.103	**0.021**(0.117)	0.002	0.962(0.962)
Entorhinal	-0.010	0.825(0.876)	0.028	0.519(0.551)
Middle temporal	-0.099	**0.029**(0.098)	-0.095	**0.026(0.040)**
Ventricular	0.101	**0.024**(0.102)	0.120	**0.004(0.014)**
Whole brain	-0.150	**0.001(0.012)**	-0.102	**0.014(0.027)**
**Cognitive measures**				
MMSE	-0.043	0.332(0.47)	-0.087	**0.035(0.050)**
CDR-SB	0.022	0.619(0.702)	0.063	0.131(0.159)
ADAS-Cog	0.044	0.318(0.541)	0.111	**0.008(0.018)**
Memory composite	-0.125	**0.005(0.041)**	-0.114	**0.006(0.017)**
EF composite	-0.075	0.093(0.263)	-0.151	**<0.001(0.001)**
Language composite	0.005	0.919(0.919)	-0.109	**0.009(0.019)**
VS	-0.043	0.327(0.506)	-0.101	**0.015(0.025)**

P values in parentheses were corrected for multiple comparisons by Benjamini-Hochberg procedure.
P values less than 0.05 were shone in bold.
Abbreviation: Aβ = β-amyloid; AD = Alzheimer's disease; ADAS-Cog = Alzheimer Disease Assessment Scale-Cognitive Subscale; CDR-SB = Clinical Dementia Rating Scale Sum of Boxes; CSF = cerebrospinal fluid; EF = executive function; FDG = fluorodeoxyglucose; MCI = mild cognitive impairment; PET = positron emission tomography; p-tau = phosphorylated tau; t-tau = total tau; MMSE = mini-mental state examination; VS = visuospatial

eTable 13. Correlations of concurrent changes in plasma p-tau181 and other measures across diagnostic groups

	CN		MCI		Dementia	
	ρ	P value	ρ	P value	ρ	P value
**CSF biomarkers**						
Aβ42	-0.020	0.727(0.823)	0.851	0.062(0.095)	0.154	0.044(0.082)
p-tau	0.093	0.094(0.177)	0.133	**0.004(0.029)**	0.212	**0.005(0.018)**
t-tau	0.099	0.076(0.162)	0.131	**0.004(0.016)**	0.194	**0.011(0.030)**
**PET imaging**						
AV45	0.164	**0.002(0.009)**	0.084	0.054(0.092)	0.021	0.779(0.827)
FDG-PET	-0.050	0.330(0.467)	-0.035	0.418(0.474)	-0.149	**0.035(0.073)**
**Structure imaging**						
Hippocampus	-0.101	0.043(0.122)	-0.031	0.459(0.488)	-0.097	0.163(0.231)
Entorhinal	0.018	0.723(0.878)	-0.006	0.894(0.894)	0.012	0.871(0.871)
Mid temporal	-0.101	0.046(0.112)	-0.084	0.052(0.056)	-0.139	0.0561(0.096)
Ventricular volumn	0.132	**0.009(0.003)**	0.123	**0.004(0.020)**	0.088	0.202(0.264)
Whole brain	-0.186	**0.001(0.002)**	-0.106	**0.012(0.041)**	-0.073	0.2862(0.348)
**Cognitive measures**						
MMSE	-0.063	0.208(0.322)	-0.083	0.050(0.122)	-0.106	0.116(0.178)
CDR-SB	0.008	0.873(0.927)	0.068	0.106(0.150)	0.068	0.313(0.354)
ADAS-Cog	0.132	**0.008(0.033)**	0.065	0.122(0.160)	0.200	**0.003(0.016)**
Memory composite	-0.195	<**0.001(0.002)**	-0.094	**0.027**(0.077)	-0.208	**0.002(0.033)**
EF composite	-0.070	0.163(0.277)	-0.137	**0.001(0.019)**	-0.156	**0.021**(0.051)
Language composite	0.008	0.879(0.879)	-0.083	0.052(0.109)	-0.194	**0.004(0.017)**
VS composite	-0.046	0.3576(0.467)	-0.042	0.316(0.384)	-0.208	**0.002(0.016)**

P values in parentheses were corrected for multiple comparisons by Benjamini-Hochberg procedure.
P values less than 0.05 were shone in bold.
Abbreviation: Aβ = β-amyloid; AD = Alzheimer's disease; ADAS-Cog = Alzheimer Disease Assessment Scale-Cognitive Subscale; CDR-SB = Clinical Dementia Rating Scale Sum of Boxes; CSF = cerebrospinal fluid; EF = executive function; FDG = fluorodeoxyglucose; MCI = mild cognitive impairment; PET = positron emission tomography; p-tau = phosphorylated tau; t-tau = total tau; MMSE = mini-mental state examination; VS = visuospatial
